# Hormonal Status May Contribute to Sex-Based Survival Differences in Epithelioid Peritoneal Mesothelioma

**DOI:** 10.1245/s10434-026-19455-x

**Published:** 2026-03-12

**Authors:** Kaelyn C. Cummins, Olivia Sears, Elio R. Bitar, Chengli Shen, Allan Tsung, Samantha M. Ruff

**Affiliations:** Department of Surgery, University of Virginia Health, Charlottesville, VA USA

**Keywords:** Peritoneal mesothelioma, Cytoreduction surgery, HIPEC, Hormone, Epithelioid, Menopause

## Abstract

**Background:**

Cytoreductive surgery (CRS) with hyperthermic intraperitoneal chemotherapy (HIPEC) is central to treating patients with epithelioid peritoneal mesothelioma (EPM). Male sex is a negative prognostic factor in mesothelioma, but the reasons for this remain unclear. We evaluated the impact of sex and age, as a proxy for menopause status, on treatment patterns and survival for EPM.

**Patients and Methods:**

Adults with unicavitary EPM were identified in the National Cancer Database (NCDB) (2006–2020). Predictors of CRS/HIPEC receipt were evaluated using logistic regression. Among patients who underwent CRS/HIPEC, variables impacting overall survival (OS) were evaluated using Cox regression. Patients were stratified by age (≤ 40 years and ≥ 60 years) into younger male, older male, premenopausal female, and postmenopausal female patients for subgroup analysis.

**Results:**

Of 1868 patients meeting inclusion criteria, 28.1% (*n* = 526) received CRS/HIPEC; of those, 286 (54.4%) were male. Male sex (odds ratio, OR 1.054, *p *= 0.015), younger age (OR 0.996, *p *< 0.001), and treatment at an academic center (OR 1.253, *p *< 0.001) were independently associated with receiving CRS/HIPEC. Males had worse OS than females after CRS/HIPEC on multivariable regression (hazard ratio, HR 1.647, *p *= 0.003). Upon age-stratification of patients who underwent CRS/HIPEC, premenopausal females had significantly improved OS compared with younger male, older male, and postmenopausal female patients on Kaplan–Meier analysis (all *p *< 0.05). Female sex significantly predicted survival in younger (HR 0.267, *p *= 0.012) but not older patients (HR 0.676, *p *= 0.061).

**Conclusions:**

Premenopausal women with EPM undergoing CRS/HIPEC had significantly improved OS compared with both males and postmenopausal females, suggesting a hormonal influence on outcomes. Future studies to elucidate the biological mechanisms underlying this sex-based disparity are necessary.

**Supplementary Information:**

The online version contains supplementary material available at 10.1245/s10434-026-19455-x.

Epithelioid peritoneal mesothelioma is a rare primary malignancy of the peritoneum for which cytoreductive surgery (CRS) combined with hyperthermic intraperitoneal chemotherapy (HIPEC) remains the cornerstone of treatment. Despite advances in surgical technique and perioperative management, survival outcomes remain heterogeneous, with male sex consistently identified as an independent prognostic indicator of worse overall survival in several large series.^1–4^ Furthermore, the recently updated Peritoneal Surface Malignancy Consortium guidelines include male sex as an intermediate-risk feature.^5^ However, the biological basis for this disparity is poorly understood.

Sex-based survival differences are increasingly recognized across solid tumors, where both immune function and hormonal milieu are implicated.^6^ In pleural mesothelioma, estrogen receptor expression (ERα versus Erβ) may modulate tumor biology and response to therapy, although direct evidence remains limited.^7^ In a small study of 52 patients with peritoneal mesothelioma, premenopausal female individuals were found to have better survival than postmenopausal female and male individuals, suggesting a possible role for sex hormones.^8^ However prior large-scale studies have not accounted for menopausal status, which could serve as a clinical surrogate for hormonal activity and may clarify the observed survival gap between male and female patients.

Given these uncertainties, we used a national cancer registry to evaluate the impact of sex and age as a proxy for menopausal status on treatment patterns and survival among patients with epithelioid peritoneal mesothelioma undergoing CRS/HIPEC. We hypothesized that premenopausal females would have improved survival compared with males and postmenopausal females, suggesting a potential role for hormones in driving sex-based disparities in peritoneal mesothelioma.

## Patients and Methods

This study was deemed exempt from informed consent by the University of Virginia Institutional Review Board owing to the deidentified nature and public availability of the dataset.

### Study Population and Groups

De-identified data were sourced from the National Cancer Database (NCDB) using the 2020 peritoneum participant user file. The NCDB is a joint project of the Commission on Cancer (CoC) of the American College of Surgeons and the American Cancer Society and encompasses more than 70% of cancer diagnoses in the USA.^9^ The NCDB and the participating hospitals are the source of the deidentified data used in this study; they have not verified and are not responsible for the validity of this study or the conclusions derived by the authors.

The study population was limited to adult (age ≥ 18 years) patients with diagnoses of peritoneal mesothelioma with epithelioid histology between 2006 and 2020. Patients with bicavitary disease were excluded. Patients were classified into CRS/HIPEC or non-CRS/HIPEC on the basis of the “Systemic/Surgery Sequence” variable in the NCDB. Patients with a value of 5 (“intraoperative systemic therapy”) or 6 (“intraoperative systemic therapy with other systemic therapy administered before or after surgery”) were considered CRS/HIPEC patients; patients with any other value for this variable were classified as non-CRS/HIPEC patients. This method of identifying patients who have undergone CRS/HIPEC has been used in several previously published studies across multiple cancer types.^10–16^

For menopausal status subgroup analysis, patients were categorized into younger male (age ≤ 40 years), older male (age ≥ 60 years), premenopausal female (age ≤ 40 years), and postmenopausal female (age ≥ 60 years). Patients between 41 and 59 years old were excluded for this portion of the analysis. These ages were chosen on the basis of a previous small study on hormonal influences on peritoneal mesothelioma^8^ and to reliably exclude women in perimenopause.^17^ Sensitivity analyses were run to confirm that our results were consistent with multiple age cutoffs in this range.

### Statistical Analysis

Univariable analysis was used to summarize baseline characteristics of patients who did and did not receive CRS/HIPEC. Continuous and categorical variables were evaluated using the *t*-test or chi-squared test, respectively. Multivariable logistic regression identified independent predictors for receipt of CRS/HIPEC.

Only patients who underwent CRS/HIPEC were included in subsequent analyses. Univariable analysis compared baseline demographic and clinical characteristics of male versus female patients. Univariable and multivariable Cox regression analysis evaluated predictors of overall survival (OS).

CRS/HIPEC is the standard of care for medically optimized patients with peritoneal epithelioid mesothelioma where complete cytoreduction can be achieved. The decision to provide neoadjuvant systemic therapy is based on high-risk features as outlined in the National Comprehensive Cancer Network (NCCN) and Peritoneal Surface Malignancy Consortium guidelines.^5,18^ In the subgroup analysis stratified by age, only patients who received CRS/HIPEC were included to focus on outcomes in optimally managed patients. Kaplan–Meier survival analysis was performed using the menopausal status variable as the predictor, with log-rank test used to compare survival curves. Subsequently, univariable Cox regression identified differences in OS between the groups. Univariable Cox regression was used to separately model OS within patients ≤ 40 years of age and in patients ≥ 60 years of age. Cause of death is not available in the NCDB; therefore, competing risks for cancer-specific survival were not able to be calculated. Results presented represent overall survival.

All statistical tests were two-sided with statistical significance demarcated by *p* < 0.05. All statistical analyses were performed using R (version 4.4.1, R Foundation for Statistical Computing, Vienna, Austria).

## Results

### Baseline Demographics and Predictors of CRS/HIPEC Receipt

A total of 1868 patients met inclusion criteria, of which 1012 (54.2%) were male and 526 (28.2%) received CRS/HIPEC. Univariable and multivariable logistic regression evaluating predictors of CRS/HIPEC receipt are summarized in Table 1. On multivariable logistic regression, male sex (odds ratio [OR] 1.054, *p* = 0.015) and younger age (OR 0.996, *p* < 0.001) were associated with undergoing CRS/HIPEC. Patients who received CRS/HIPEC traveled farther for treatment (OR 1.0002, *p* < 0.001) and were significantly less likely to be treated at a nonacademic hospital compared with an academic center (OR 0.798, *p* < 0.001). Notably, treatment year was not a significant predictor of CRS/HIPEC receipt (*p* = 0.62), suggesting that use of CRS/HIPEC for this cancer has not significantly increased over 2006–2020. Other clinical and socioeconomic factors including race, ethnicity, zip code education or income level, were not independent predictors of CRS/HIPEC receipt.

**Table 1:** Baseline demographics by HIPEC receipt and multivariable logistic regression for predictors of HIPEC receipt; all adult patients with epithelioid peritoneal mesothelioma without bicavitary disease from 2006–2020

	Univariable			Multivariable		
	Non-HIPEC (*n* = 1342)	HIPEC (*n* = 526)	*p*-Value	HR	95% CI	*p*-Value
*Sex*			0.026			
Male	705 (52.5%)	307 (58.4%)		1.054		0.015
Female	637 (47.5%)	219 (41.6%)				Baseline
Age (years; mean ± SD)	62.1 ± 14.5	56.3 ± 13.7	< 0.0001	0.996		0.0003
*Race*			0.82			
White	1200 (89.4%)	468 (89.0%)				
Black	77 (5.7%)	24 (4.6%)				
AAPI	44 (3.3%)	17 (3.2%)				
Other	12 (0.9%)	4 (0.7%)				
Unknown	9 (0.7%)	13 (2.5%)				
*Ethnicity*			0.25			
Hispanic	98 (7.3%)	30 (5.7%)				
Non-Hispanic	1205 (89.8%)	484 (92.0%)				
Unknown	39 (2.9%)	12 (2.3%)				
*Region*			< 0.0001			
Midwest	329 (24.5%)	99 (18.8%)				Baseline
Northeast	320 (23.8%)	167 (31.7%)		0.984		0.60
South	405 (30.2%)	151 (28.7%)		1.030		0.30
West	178 (12.3%)	45 (8.6%)		0.973		0.45
Unknown	110 (8.2%)	64 (12.2%)				
*Education quartile*			0.43			
Q1	183 (13.6%)	68 (12.9%)				
Q2	295 (22.0%)	89 (16.9%)				
Q3	340 (25.3%)	127 (24.1%)				
Q4	345 (36.1%)	133 (25.3%)				
Unknown	179 (13.3%)	109 (20.7%)				
Income quartile			0.10			
Q1	149 (11.1%)	51 (9.7%)				
Q2	249 (18.6%)	84 (16.0%)				
Q3	279 (20.8%)	80 (15.2%)				
Q4	484 (36.1%)	200 (38.0%)				
Unknown	181 (13.5%)	111 (21.1%)				
Population density			0.82			
Metropolitan	1063 (79.2%)	388 (73.8%)				
Urban	173 (12.9%)	60 (11.4%)				
Rural	20 (1.5%)	9 (1.7%)				
Unknown	86 (6.4%)	69 (13.1%)				
Distance from treatment center (miles; mean ± SD)	62.7 ± 204	224.0 ± 470	< 0.0001	1.0002		< 0.0001
*Charlson–Deyo score*			0.01			
0	971 (72.4%)	410 (77.9%)				Baseline
1	246 (18.3%)	86 (16.3%)		0.962		0.17
2	75 (5.6%)	23 (4.4%)		0.971		0.53
3+	50 (3.7%)	7 (1.3%)		0.897		0.07
*Year of diagnosis*			0.62			
2006-2010	330 (24.6%)	120 (22.8%)				
2011-2015	455 (33.9%)	189 (35.9%)				
2016-2020	557 (41.5%)	217 (41.3%)				
*Facility type*			<0.0001			
Academic/research	551 (41.1%)	356 (67.7%)				Baseline
Other	791 (58.9%)	170 (32.3%)		0.798		< 0.0001
*Primary payor*			< 0.0001			
Private insurance	575 (42.8%)	314 (59.7%)				Baseline
Government insurance	704 (52.5%)	195 (37.1%)		0.985		0.56
Uninsured	30 (2.2%)	11 (2.1%)		0.947		0.49
Unknown	33 (2.5%)	6 (1.1%)				
*Lymph node positivity*			0.005			
Negative	1269 (94.6%)	478 (90.9%)				Baseline
Positive	73 (5.4%)	48 (9.1%)		0.985		0.76
Grade^†^			0.019			
G1	98 (7.3%)	39 (7.4%)				
G2	36 (2.7%)	31 (5.9%)				
G3	59 (4.4%)	22 (4.2%)				
Unknown	1149 (85.6%)	434 (82.5%)				
*Lymphovascular invasion*^*†*^			0.40			
Negative	103 (7.7%)	79 (15.0%)				
Positive	28 (2.1%)	15 (2.9%)				
Unknown	1211 (90.2%)	432 (82.1%)				

^†^Grade and lymphvascular invasion not included in multivariable analysis owing to large number of missing values

AAPI Asian American and Pacific Islander

Of the patients who underwent CRS/HIPEC, 307 (58.4%) were male and 219 (41.6%) were female. Baseline characteristics of this group, stratified by sex, are summarized in Table 2. Female patients were more likely to be younger (female: 53.3 ± 13.7 years, male: 58.5 ± 13.3 years, *p* < 0.001) and to have private insurance (female: 66.2%, male: 55.0%, *p* = 0.03) compared with males. Other sociodemographic variables were similar between the sexes.

**Table 2:** Baseline demographics in patient by sex; all adult patients with epithelioid peritoneal mesothelioma without bicavitary disease who underwent CRS/HIPEC from 2006 to 2020

	Females (*n* = 219)	Males (*n* = 307)	*p*-Value
Age (years; mean ± SD)	53.3 ± 13.7	58.5 ± 13.3	< 0.0001
*Race*			0.44
White	188 (85.8%)	280 (91.2%)	
Black	11 (5.0%)	13 (4.2%)	
AAPI	10 (4.6%)	7 (2.3%)	
Other	2 (0.9%)	2 (0.7%)	
Unknown	8 (3.7%)	5 (1.6%)	
*Ethnicity*			0.14
Hispanic	13 (5.9%)	17 (5.5%)	
Non-Hispanic	198 (90.4%)	286 (93.2%)	
Unknown	8 (3.7%)	4 (1.3%)	
*Region*			0.34
Midwest	35 (16%)	64 (20.8%)	
Northeast	66 (30.1%)	101 (32.9%)	
South	68 (31.1%)	83 (27.0%)	
West	15 (6.8%)	30 (9.8%)	
Unknown	35 (16.0%)	29 (9.4%)	
*Education quartile*			0.97
Q1	27 (12.3%)	41 (13.4%)	
Q2	36 (16.4%)	53 (17.3%)	
Q3	52 (23.7%)	75 (24.4%)	
Q4	57 (26.0%)	76 (24.8%)	
Unknown	47 (21.5%)	62 (20.2%)	
*Income quartile*			0.19
Q1	17 (7.8%)	34 (11.1%)	
Q2	29 (13.2%)	55 (17.9%)	
Q3	38 (17.4%)	42 (13.7%)	
Q4	88 (40.2%)	112 (36.5%)	
Unknown	47 (21.5%)	64 (20.8%)	
*Population density*			0.33
Metropolitan	172 (78.5%)	216 (70.4%)	
Urban	21 (9.6%)	39 (12.7%)	
Rural	3 (1.4%)	6 (2.0%)	
Unknown	23 (10.5%)	46 (15.0%)	
Great circle distance (miles; mean ± SD)	223 ± 465	224 ± 475	0.98
Charlson–Deyo score			0.45
0	168 (76.7%)	242 (78.8%)	
1	36 (16.4%)	50 (16.3%)	
2	13 (5.9%)	10 (3.3%)	
3+	2 (0.9%)	5 (1.6%)	
*Year of diagnosis*			0.22
2006–2010	46 (21.0%)	74 (24.1%)	
2011–2015	73 (33.3%)	116 (37.8%)	
2016–2020	100 (45.7%)	117 (38.1%)	
*Facility type*			0.37
Academic/research	143 (65.3%)	213 (69.4%)	
Other	76 (34.7%)	94 (30.6%)	
*Primary payor*			0.030
Private insurance	145 (66.2%)	169 (55.0%)	
Government insurance	68 (31.1%)	127 (41.4%)	
Uninsured	6 (2.7%)	5 (1.6%)	
Unknown	0 (0%)	6 (2.0%)	
*Lymph node positivity*			1
Negative	199 (90.9%)	279 (90.9%)	
Positive	20 (9.1%)	28 (9.1%)	
*Grade*			0.47
G1	18 (8.2%)	21 (6.8%)	
G2	10 (4.6%)	21 (6.8%)	
G3	8 (3.7%)	14 (4.6%)	
Unknown	183 (83.6%)	251 (81.8%)	
*Lymphovascular invasion*			0.73
Negative	30 (13.7%)	49 (16.0%)	
Positive	7 (3.2%)	8 (2.6%)	
Unknown	182 (83.1%)	250 (81.4%)	

AAPI Asian American and Pacific Islander

A subgroup of female patients of age < 75 years and Charlson–Deyo score of 0 or 1 was generated to represent the lowest-risk population that can be examined under the constraints of the NCDB. Of this subgroup (*n* = 692), only 197 (28.5%) patients underwent CRS/HIPEC.

### Overall Survival in CRS/HIPEC Group

**Fig. 1:**
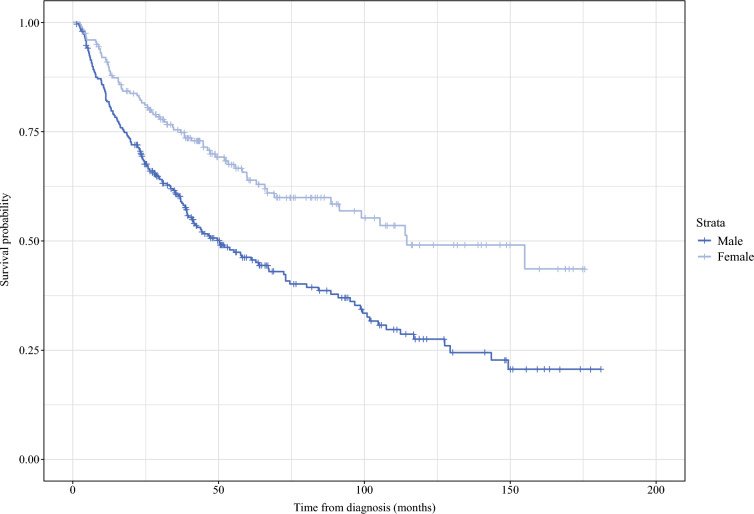
Kaplan–Meier of overall survival in patients who underwent CRS/HIPEC, stratified by sex

Kaplan–Meier analysis (Fig. 1) and multivariable Cox regression were used to evaluate OS in patients who received CRS/HIPEC (Table 3). While female sex had been associated with decreased likelihood of undergoing CRS/HIPEC, it was also significantly associated with better OS (hazard ratio [HR] 0.635, *p *= 0.008) than male sex. Older age (HR 1.028, *p* < 0.001) and South or Midwest location (reference: West; South: HR 2.415, *p *= 0.045; Midwest: HR 3.455, *p *= 0.005) were also independently associated with worse OS in patients who received CRS/HIPEC.

**Table 3:** Univariable and multivariable Cox regressions on survival in adult CRS/HIPEC population

	Univariable analysis			Multivariable analysis		
Variable	HR	95% CI	*p*-Value	HR	95% CI	*p*-Value
Female sex	0.538	0.408, 0.708	< 0.0001	0.635	0.452, 0.890	0.008
Age	1.029	1.019, 1.039	< 0.0001	1.028	1.010, 1.046	0.003
*Race (baseline: white)*						
Black	1.029	0.528, 2.005	0.93			
AAPI	0.498	0.159, 1.559	0.23			
Other	0.244	0.034, 1.744	0.16			
Non-Hispanic ethnicity	1.556	0.798, 3.029	0.19			
*Region (baseline: West)*						
Northeast	1.909	0.990, 3.681	0.054	2.213	0.944, 5.187	0.068
South	1.776	0.912, 3.459	0.09	2.415	1.021, 5.711	0.045
Midwest	2.758	1.404, 5.416	0.003	3.455	1.460, 8.177	0.005
*Education quartiles (baseline: highest)*						
Q2	1.291	0.767, 2.173	0.34			
Q3	1.157	0.713, 1.875	0.56			
Q4	1.351	0.838, 2.179	0.22			
*Income quartiles (baseline: highest)*						
Q2	1.895	1.057, 3.398	0.032	2.059	1.096, 3.869	0.025
Q3	1.445	0.805, 2.592	0.22	1.752	0.939, 3.269	0.078
Q4	1.435	0.842, 2.446	0.18	1.558	0.883, 2.834	0.12
*Population density (baseline: metropolitan)*						
Urban	1.363	0.947, 1.961	0.10			
Rural	0.802	0.255, 2.515	0.70			
Distance from treatment center	0.9997	0.9993, 1.000	0.09			
*Charlson–Deyo score (baseline: 0)*						
1	1.302	0.947, 1.79	0.11			
2	0.691	0.325, 1.47	0.34			
3+	2.365	0.755, 7.41	0.14			
*Year of diagnosis (baseline: 2006–2010)*						
2011–2015	1.224	0.905, 1.654	0.19			
2016–2020	0.748	0.515, 1.087	0.13			
Facility type (baseline: academic)	0.920	0.696, 1.216	0.56			
*Primary payor (baseline: private)*						
Government	1.562	1.202, 2.031	0.0009	1.046	0.707, 1.548	0.82
Uninsured	1.158	0.428, 3.133	0.77	2.166	0.784, 5.987	0.14
Positive lymph nodes	1.300	0.858, 1.971	0.22			
*Grade (baseline: G1)*^*†*^						
G2	3.023	1.352, 6.760	0.007			
G3	6.496	2.676, 15.77	<0.0001			
Positive lymphovascular invasion	0.975	0.410, 2.319	0.95			

^†^Grade not included in multivariable analysis owing to large number of missing values

*AAPI* Asian American and Pacific Islander

### Subgroup Analysis by Hormonal Status

**Fig. 2:**
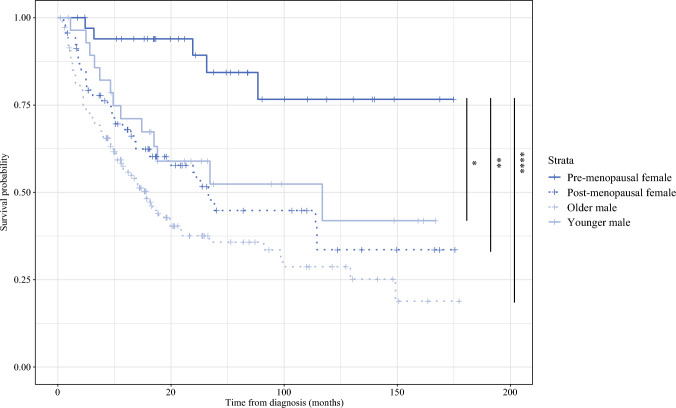
Kaplan–Meier of overall survival in patients who underwent CRS/HIPEC, stratified by sex and age to represent presumed menopausal status; **p < 0.01, ****p < 0.0001; all other pairwise comparisons nonsignificant; *p*-values corrected for multiple comparisons

Upon stratification by sex and age, as a proxy for menopausal status, there were 31 younger male, 154 older male, 76 postmenopausal female, and 41 premenopausal female patients who received CRS/HIPEC, with patients aged 41–59 years excluded. On Kaplan–Meier analysis, median survival was not reached for premenopausal females at 175 months (Fig. 2). Premenopausal females had significantly greater median survival than postmenopausal females (66.6 months, *p *= 0.001), younger males (116.9 months, *p *= 0.014), and older males (39.1 months, *p *< 0.001).

On univariable Cox regression analysis, premenopausal females had significantly improved OS compared with postmenopausal females (HR 4.557, *p *= 0.002), younger males (HR 3.676, *p *= 0.013), and older males (HR 6.751, *p *< 0.001). This remained consistent on multivariable Cox regression (Table 4), with premenopausal females continuing to have improved OS compared with postmenopausal females (HR 4.125, *p *= 0.004), older males (HR 6.012, *p *< 0.001), and younger males (HR 4.174, *p *= 0.007). These results were consistent when tested across multiple age cutoffs in a sensitivity analysis. Additionally, peri-menopausal women (age 41–59 years) had survival that was intermediate between the pre- and postmenopausal women (data not shown).

**Table 4:** Univariable and multivariable Cox regression on overall survival in CRS/HIPEC population by sex/age composite variable; patients aged 41–59 years excluded

	Univariable analysis			Multivariable analysis		
Variable	HR	95% CI	*p*-Value	HR	95% CI	*p*-Value
*Hormone status (baseline: premenopausal females)*						
Postmenopausal females	4.557	1.774, 11.71	0.002	4.125	1.587, 10.72	0.004
Older males	6.751	2.732, 16.68	< 0.0001	6.012	2.386, 15.15	0.0001
Younger males	3.676	1.310, 10.31	0.013	4.174	1.485, 11.73	0.007
*Race (baseline: white)*						
Black	0.688	0.219, 2.163	0.52			
AAPI	0.512	0.219, 2.163	0.35			
Other	0.253	0.035, 1.817	0.17			
Non-Hispanic ethnicity	1.860	0.687, 5.036	0.22			
*Education quartiles (baseline: highest)*						
Q2	0.822	0.413, 1.634	0.58			
Q3	0.815	0.423, 1.562	0.54			
Q4	1.138	0.604, 2.144	0.69			
*Income quartiles (baseline: highest)*						
Q2	1.789	0.844, 3.792	0.13			
Q3	1.096	0.513, 2.343	0.81			
Q4	1.329	0.674, 2.618	0.41			
*Population density (baseline: metropolitan)*						
Urban	1.265	0.802, 1.996	0.31			
Rural	0.683	0.168, 2.775	0.59			
Distance from treatment center	0.9995	0.9991, 1	0.066			
Charlson–Deyo score (baseline: 0)						
1	1.370	0.914, 2.055	0.13			
2	0.479	0.152, 1.514	0.21			
3+	2.187	0.692, 6.910	0.18			
*Year of diagnosis (baseline: 2006–2010)*						
2011–2015	1.212	0.803, 1.827	0.36			
2016–2020	0.772	0.481, 1.238	0.28			
Facility type (baseline: academic)	0.749	0.523, 1.074	0.12			
*Primary payor (baseline: private)*						
Government	1.652	1.160, 2.354	0.005	1.244	0.846, 1.829	0.27
Uninsured	< 0.0001	0, Inf	0.99	< 0.0001	0, Inf	0.99
Positive lymph nodes	0.857	0.449, 1.637	0.64			
*Grade (baseline: G1)*^*†*^						
G2	1.577	0.444, 5.599	0.48			
G3	3.049	0.857, 10.84	0.09			
Positive lymphovascular invasion	0.711	0.210, 2.411	0.58			

^†^Grade not included in multivariable analysis owing to large number of missing values

AAPI Asian American and Pacific Islander, Inf infinity

Subsequently, OS in younger patients and older patients was analyzed separately using univariable Cox regression. In younger patients (Supplementary Table 1), female sex emerged as the only significant predictor of OS (HR 0.267, *p *= 0.012); however, in older patients (Supplementary Table 2), female sex did not reach statistical significance as a predictor of OS (HR 0.676, *p *= 0.061).

## Discussion

Female patients with peritoneal mesothelioma consistently demonstrate better survival than male patients, but the underlying reason is not well understood. Small studies have suggested that hormonal factors may play a role. This is the first study to apply a large national database to specifically examine the influence of sex and presumed menopause status on outcomes in patients with epithelioid peritoneal mesothelioma treated with CRS/HIPEC. Consistent with prior studies,^3,19–21^ female patients in our cohort had significantly improved OS compared with male patients, despite male sex being an independent predictor of CRS/HIPEC receipt in our population. Other predictors of CRS/HIPEC receipt, including younger age, treatment at academic centers, and greater travel distance, are consistent with prior literature.^3,20^

CRS/HIPEC is the first-line treatment for low-risk patients with epithelioid peritoneal mesothelioma.^5,21^ Despite this, a relatively small fraction (28.2%) of the patients in our cohort underwent CRS/HIPEC. Among female patients under 75 years of age with epithelioid histology and a Charlson–Deyo score of 0 or 1, the lowest-risk population that can be examined under the constraints of NCDB variables, only 28.5% of patients actually underwent CRS/HIPEC. While there are several uncaptured variables that also contribute to risk determination, such as Ki-67 score, this suggests that CRS/HIPEC may be underutilized in a patient population that could significantly benefit from its use. This is consistent with results from an NCDB analysis of peritoneal mesothelioma of all histologies.^21^ Unfortunately, our results suggest that the proportion of patients undergoing CRS/HIPEC has not significantly increased over the time period of our study. This may be related to the rarity of peritoneal mesothelioma and resultant lack of awareness of diagnosis and treatment protocols, especially in nonacademic hospitals that less frequently encounter patients with this malignancy.

Additionally, it remains unclear why male patients were more likely to receive CRS/HIPEC in this study. Prior literature suggests that female patients often present with more favorable disease characteristics, including lower peritoneal cancer index (PCI), lower Ki-67, and more favorable histologic features.^1,22,23^ These findings would suggest that females may be better candidates for CRS/HIPEC; however, we were unable to confirm these differences within our NCDB cohort owing to missing data. One multi-institutional study reported a higher likelihood of CRS/HIPEC utilization among females,^1^ although this international cohort may not reflect practice patterns in the USA. Further research is warranted to determine whether CRS/HIPEC is underutilized among female patients in the USA and to identify the factors driving the observed sex-based differences in treatment utilization.

Subgroup analysis showed that younger female patients had the best OS compared with other cohorts. Using age as a proxy for menopause status, this suggests that hormonal differences may contribute to the known sex disparity in peritoneal mesothelioma. To date, only small studies have demonstrated differences on the basis of presumed menopausal status.^8^ By leveraging a large and robust database, we confirmed this association and found that it could not be explained by other clinicodemographic or tumor factors.

Studies focusing on the effect of sex hormones in peritoneal mesothelioma are limited. The studies that have been conducted have largely focused on estrogen receptor (ER), which encompasses both α and β subtypes and is known to be a prognostic factor in several other cancers, including breast, lung, colorectal, and ovarian.^24–28^ Specifically, research in other cancers suggests that ERβ expression has a protective effect compared with ERα expression, although these data are controversial, and the impact can vary by the cellular location of ER.^25–27^ When evaluated in pleural mesothelioma, increased ERβ expression, compared with ERα, was associated with improved survival.^7^ In a study on peritoneal mesothelioma, cytoplasmic ERβ expression on immunohistochemistry was associated with worse OS compared with nuclear ERβ expression.^29^ However, regardless of ERβ expression localization, female patients still demonstrated improved OS compared with males in this study. Furthermore, ERα expression was only detected in 9% of patients. Another small case series examining the expression of sex hormone receptors in 20 patients with peritoneal mesothelioma showed that 80% of tumors were positive for ER, 100% were positive for progesterone receptor (PR), and 65% were positive for androgen receptor (AR).^23^ ER and PR expression was higher in female patients, while AR expression was similar between female and male patients.

This study has several limitations. First, large database-driven retrospective studies introduce inherent risk of selection bias and unmeasured confounding variables, as well as the risk of missing patients who were not appropriately coded as having received intraoperative chemotherapy. Moreover, the NCDB is limited in terms of granularity of the data it provides; while we stratified by sex and age as surrogates for menopausal and by extension hormonal status, direct measures of menopausal status, reproductive history including oophorectomy, and exogenous hormone use (such as oral contraceptive use or hormone replacement therapy) were not available. As such, we acknowledge that the use of age cutoffs alone for classification of women as pre- and postmenopausal may introduce some degree of misclassification and may not capture the full extent of hormonal influences. Second, important clinical variables including Ki-67 and thrombocytosis, which are considered high-risk criteria and therefore influence treatment decisions, are not available for peritoneal mesothelioma in the NCDB. Additionally, treatment-related variables, such as medication selection and dosing, during CRS/HIPEC were not available and may impact survival outcomes, especially given the known heterogeneity of HIPEC treatment regimens.^5,30^ The NCDB also does not include cause of death, which prevented us from evaluating cancer-specific mortality or running a competing risks analysis. Given that older patients are more likely to die of non-cancer-related causes than younger patients, this could introduce bias into our results. Finally, because the NCDB only collects data from hospitals accredited by the Commission on Cancer (CoC), our findings may be less generalizable to patients treated at nonacademic or lower-volume centers.

Despite these limitations, our study highlights important sex- and age-related differences in outcomes after CRS/HIPEC for epithelioid peritoneal mesothelioma. Although limited in granularity, the NCDB does capture long-term survival, allowing us to detect clinically meaningful differences across subgroups. Given the rarity of epithelioid peritoneal mesothelioma, large national databases such as the NCDB are needed to gather sufficiently large cohorts to allow for meaningful survival analyses and direct future areas of study. The NCDB provides the largest national cohort of patients with epithelioid peritoneal mesothelioma treated with CRS/HIPEC, enabling robust subgroup comparisons that would not be feasible in smaller institutional series. The long study period and broad geographic representation further strengthen the validity and applicability of our findings, even if several granular treatment details are lacking. Importantly, the consistent survival advantage observed among premenopausal women, especially when compared with men of a similar age group, and the absence of a survival advantage when comparing older female and older male patients, suggests a biologically meaningful signal that is unlikely to be explained entirely by missing data.

In patients with peritoneal mesothelioma with epithelioid histology, female sex is associated with improved survival among all patients and those treated with CRS/HIPEC. This survival advantage may partially be driven by premenopausal women, who have significantly better OS when compared with postmenopausal women, older men, and younger men. This suggests that sex hormones may play a role in the underlying cancer biology and outcomes of peritoneal mesothelioma. Further research is needed to clarify the impact of various hormones on development, progression, and treatment of peritoneal mesothelioma and should focus on prospective, multicenter studies incorporating biologic markers of sex hormone status to more precisely evaluate their role in epithelioid peritoneal mesothelioma. Molecular studies investigating hormone receptor expression and signaling pathways in tumor tissue may further clarify the underlying mechanisms of the observed sex- and age-related survival differences.

## Supplementary Information

Below is the link to the electronic supplementary material.

Supplementary file1 (DOCX 15 kb)
