# Supplementary material for: Hormonal Status May Contribute to Sex-Based Survival Differences in Epithelioid Peritoneal Mesothelioma
Source: Ann Surg Oncol. 2026 Mar 12;33(6):5304–14. doi: 10.1245/s10434-026-19455-x (PMC13033236; doi:10.1245/s10434-026-19455-x)
Supplement: Supplementary file 1 — Supplementary file1 (DOCX 15 kb) [file 10434_2026_19455_MOESM1_ESM.docx]

|  | **Univariable analysis** | | |
| --- | --- | --- | --- |
| **Variable** | **HR** | **95% CI** | **P-value** |
| Female sex | 0.267 | 0.095, 0.749 | 0.012 |
| Age | 1.074 | 0.982, 1.174 | 0.12 |
| Race (baseline: White) |  |  |  |
| Black | 3.665 | 0.461, 29.15 | 0.22 |
| AAPI | <0.0001 | 0, inf | 1.0 |
| Other | <0.0001 | 0, inf | 1.0 |
| Non-Hispanic ethnicity | 0.681 | 0.155, 2.999 | 0.61 |
| Education quartiles (baseline: highest) |  |  |  |
| Q2 | <0.0001 | 0, inf | 1.0 |
| Q3 | 0.848 | 0.202, 3.561 | 0.82 |
| Q4 | 1.046 | 0.260, 4.204 | 0.95 |
| Income quartiles (baseline: highest) |  |  |  |
| Q2 | 1.479 | 0.238, 9.181 | 0.67 |
| Q3 | <0.0001 | 0, inf | 1.0 |
| Q4 | 1.844 | 0.396, 8.579 | 0.44 |
| Population density (baseline: metropolitan) |  |  |  |
| Urban | 0.459 | 0.060, 3.510 | 0.45 |
| Rural | 1.628 | 0.207, 12.82 | 0.64 |
| Distance from treatment center | 0.9999 | 0.9989, 1.001 | 0.80 |
| Charlson-Deyo score (baseline: 0) |  |  |  |
| 1 | 2.154 | 0.766, 6.058 | 0.15 |
| 2 | NA | NA | NA |
| 3+ | NA | NA | NA |
| Year of diagnosis (baseline: 2006-2010) |  |  |  |
| 2011-2015 | 0.619 | 0.205, 1.873 | 0.40 |
| 2016-2020 | 1.084 | 0.261, 3.265 | 0.90 |
| Facility type (baseline: academic) | 1.982 | 0.263, 14.95 | 0.51 |
| Primary payor (baseline: private) |  |  |  |
| Government | 1.679 | 0.549, 5.137 | 0.36 |
| Uninsured | <0.0001 | 0, inf | 1.0 |
| Positive lymph nodes | 2.034 | 0.659, 6.277 | 0.22 |

Supplementary Table 1: Univariable Cox regression for overall survival in young patients (age≤40), *n* = 72. AAPI: Asian American and Pacific Islander.

|  | **Univariable analysis** | | |
| --- | --- | --- | --- |
| **Variable** | **HR** | **95% CI** | **P-value** |
| Female sex | 0.676 | 0.450, 1.018 | 0.061 |
| Age | 1.022 | 0.991, 1.053 | 0.17 |
| Race (baseline: White) |  |  |  |
| Black | 0.444 | 0.109, 1.801 | 0.26 |
| AAPI | 0.641 | 0.158, 2.601 | 0.53 |
| Other | 0.372 | 0.051, 2.688 | 0.33 |
| Non-Hispanic ethnicity | 2.389 | 0.589, 9.694 | 0.22 |
| Region (baseline: West) |  |  |  |
| Northeast | 2.203 | 0.875, 5.547 | 0.094 |
| South | 2.003 | 0.776, 5.174 | 0.15 |
| Midwest | 3.166 | 1.221, 8.206 | 0.018 |
| Education quartiles (baseline: highest) |  |  |  |
| Q2 | 0.831 | 0.392, 1.765 | 0.63 |
| Q3 | 0.684 | 0.327, 1.430 | 0.31 |
| Q4 | 1.034 | 0.505, 2.115 | 0.93 |
| Income quartiles (baseline: highest) |  |  |  |
| Q2 | 1.888 | 0.825, 4.322 | 0.13 |
| Q3 | 1.113 | 0.489, 2.533 | 0.80 |
| Q4 | 1.119 | 0.525, 2.384 | 0.77 |
| Population density (baseline: metropolitan) |  |  |  |
| Urban | 1.298 | 0.810, 2.081 | 0.28 |
| Rural | 0.543 | 0.075, 3.903 | 0.54 |
| Distance from treatment center | 0.9995 | 0.9990, 1 | 0.10 |
| Charlson-Deyo score (baseline: 0) |  |  |  |
| 1 | 1.181 | 0.759, 1.836 | 0.46 |
| 2 | 0.369 | 0.117, 1.169 | 0.09 |
| 3+ | 1.705 | 0.538, 5.404 | 0.36 |
| Year of diagnosis (baseline: 2006-2010) |  |  |  |
| 2011-2015 | 1.252 | 0.796, 1.968 | 0.33 |
| 2016-2020 | 0.667 | 0.398, 1.117 | 0.12 |
| Facility type (baseline: academic) | 1.394 | 0.908, 2.139 | 0.13 |
| Primary payor (baseline: private) |  |  |  |
| Government | 1.200 | 0.809, 1.780 | 0.36 |
| Uninsured | <0.0001 | 0, Inf | 1.0 |
| Positive lymph nodes | 0.769 | 0.337, 1.755 | 0.53 |

Supplementary Table 2: Univariable Cox regression for overall survival in older patients (age ≥ 60), *n* = 230. AAPI: Asian American and Pacific Islander.
